# Microbiota injury during conditioning for allogeneic hematopoietic stem cell transplantation

**DOI:** 10.25122/jml-2026-0065

**Published:** 2026-05

**Authors:** Lavinia-Eugenia Lipan, Karina-Doris Vihta, Dumitru Jardan, Andi Palade, Iuliana Iordan, Alexandra Marcoci, Oana-Gabriela Craciun, Alina Daniela Tănase

**Affiliations:** 1 Carol Davila University of Medicine and Pharmacy, Bucharest, Romania; 2 Fundeni Clinical Institute, Bucharest, Romania; 3 MedLife Romania, Bucharest, Romania; 4 National Registry of Voluntary Hematopoietic Stem Cell Donors, Bucharest, Romania

**Keywords:** allogeneic hematopoietic stem cell transplantation, gut microbiota, conditioning regimen, Shannon diversity, 16S rRNA sequencing, dysbiosis, total body irradiation, allo-HSCT, allogeneic hematopoietic stem cell transplantation, aGVHD, acute graft-versus-host disease, ALL, acute lymphoblastic leukemia, AML, acute myeloid leukemia, ASV, amplicon sequence variant, CFA, cyclophosphamide, CMBI, conditioning-associated microbiota injury, CNIs, calcineurin inhibitors, CONSORT, Consolidated Standards of Reporting Trials, FLU, fludarabine, HAPLO, haploidentical donor, IQR, interquartile range, MAC, myeloablative conditioning, MEL, melphalan, MMF, mycophenolate mofetil, MMUD, mismatched unrelated donor, MRD, matched related donor, MTX, methotrexate, MUD, matched unrelated donor, NMA, non-myeloablative conditioning, PBSC, peripheral blood stem cells, PCoA, principal coordinates analysis, PERMANOVA, permutational multivariate analysis of variance, PTCy, post-transplant cyclophosphamide, Q1–Q3, first to third quartile, RIC, reduced-intensity conditioning, rRNA, ribosomal ribonucleic acid, T0, pre-conditioning timepoint (admission), T1, day of graft infusion (post-conditioning), TBI, total body irradiation, TT, thiotepa

## Abstract

Allogeneic hematopoietic stem cell transplantation (allo-HSCT) is a potentially curative treatment for hematological malignancies, but it is associated with substantial gut microbial dysbiosis linked to graft-versus-host disease, infections, and transplant-related mortality. The conditioning regimen represents the first major iatrogenic insult delivered within the transplant pathway, but its specific contribution to microbiota injury, independent of subsequent aplasia, antibiotic exposure, and mucositis, remains incompletely characterized. We conducted a prospective, single-center, observational longitudinal study at Fundeni Clinical Institute between August 2024 and February 2025, analyzing paired stool samples collected before conditioning (T0) and on the day of stem cell infusion (T1) from 47 adult allo-HSCT recipients. Microbial diversity was assessed using 16S rRNA gene sequencing (V4–V5 region), with the Shannon diversity index as the primary outcome. Statistical analyses were performed using SPSS, including Wilcoxon signed-rank tests for paired comparisons, Mann–Whitney U tests for between-group differences, and Kruskal–Wallis H tests. Shannon diversity decreased substantially between T0 and T1 (median 4.91 vs. 3.51; Wilcoxon *P* < 0.001; effect size r = 0.62), with 37 of 47 patients (78.7%) showing decline. This conditioning-induced injury was reproducible across three stratification dimensions: agent type (chemotherapy vs. TBI-containing; *P* = 0.67), intensity (RIC vs. MAC; *P* = 0.92), and number of cytotoxic agents (2 vs. 3; *P* = 0.54), with statistically significant within-subgroup decline observed in the four most represented regimens. Beta diversity analysis revealed a significant shift in community composition (PERMANOVA *P* < 0.001) without differential dispersion. Conditioning produces a substantial, biologically coherent microbiota injury before aplasia-associated factors come into play, but further studies on large cohorts are needed.

## Introduction

Allogeneic hematopoietic stem cell transplantation (allo-HSCT) is a potentially curative treatment for a broad spectrum of hematological malignancies. Despite continuing improvements in supportive care and donor selection, transplant recipients remain at substantial risk of morbidity and mortality for graft-versus-host disease (GVHD), opportunistic infections, and transplant-related complications [1]. Over the past decade, accumulating evidence has identified the intestinal microbiota as a clinically relevant determinant of outcomes after allo-HSCT, with a central role in immune modulation, mucosal barrier integrity, and colonization resistance against pathogenic organisms [2].

Landmark multicenter studies have demonstrated that loss of microbial diversity during the peritransplant period independently predicts transplant-related mortality and acute GVHD [3,4]. The seminal work by Peled and colleagues, analyzing 8,767 stool samples from 1,362 patients across four institutions, established the intestinal microbiota at the time of engraftment as a robust biomarker of overall survival, independent of established clinical risk factors [3]. These findings have prompted the development of strategies to protect the microbiota, including narrow-spectrum antibiotic protocols, dietary modifications, prebiotic supplementation, and engineered microbial consortia [5-7].

The peritransplant period exposes the gut microbiota to multiple sequential insults: cumulative pre-transplant antibiotic and antineoplastic exposures, conditioning regimen, post-conditioning aplasia, broad-spectrum antibiotic prophylaxis, mucositis, and altered nutrition [8,9]. Among these, the conditioning regimen represents the first major iatrogenic insult delivered within a controlled, time-defined therapeutic window. Conditioning combines high-dose cytotoxic chemotherapy, often with or without total body irradiation (TBI), producing direct mucosal toxicity, alterations in gut motility, and indirect effects through metabolic and immune-modulatory mechanisms [10].

Shouval and colleagues, in a retrospective analysis of 1,188 allo-HSCT recipients, developed the conditioning-associated microbiota injury (CMBI) grading system, which stratifies regimens into three injury tiers based on alpha-diversity loss; this work established that high-intensity regimens such as TBI-thiotepa-cyclophosphamide cause the greatest disruption, whereas non-myeloablative fludarabine-cyclophosphamide with low-dose TBI causes minimal injury [11]. Jørgensen *et al*., using shotgun metagenomic sequencing in 254 patients, further demonstrated that myeloablative regimens are associated with both greater taxonomic disruption and broader loss of microbial metabolic functions, providing a functional substrate for downstream clinical consequences [12]. More recently, Rashidi and colleagues compared TBI- versus chemotherapy-based myeloablative conditioning, identifying regimen-specific signatures of oral and gut microbiota injury that persist long after the acute transplant period [13].

Despite these advances, several knowledge gaps remain. First, most prior studies have characterized microbiota injury at engraftment or beyond, conflating the effects of conditioning with those of subsequent aplasia, antibiotic exposure, and mucositis. Second, the relative contribution of different conditioning modalities — particularly in cohorts dominated by reduced-intensity regimens that reflect contemporary clinical practice — has not been comprehensively assessed in single-institution prospective cohorts with consistent sample processing. Third, the day of graft infusion (T1), occurring immediately after completion of conditioning but before the onset of aplasia and systemic broad-spectrum antibacterial prophylaxis, represents a biologically critical time point that has been underexplored as a discrete window for diversity assessment and potential intervention.

In this prospective single-center cohort study, we characterized the immediate impact of conditioning on gut microbial diversity by comparing paired stool samples collected at admission, before any conditioning agent (T0), with samples collected on the day of graft infusion, immediately after completion of conditioning and before the onset of post-transplant aplasia (T1). We assessed the magnitude and reproducibility of the conditioning-induced microbiota injury across multiple stratification dimensions, including regimen type, intensity, and number of cytotoxic agents.

## Material and Methods

### Study design and population

**Figure 1:**
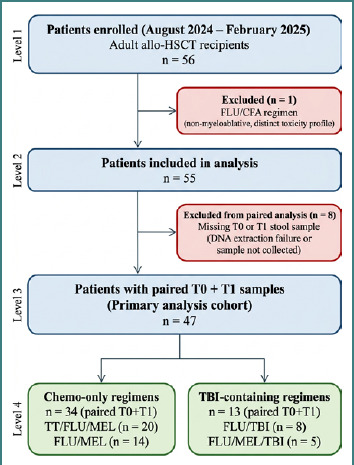
CONSORT-style flow diagram of patient inclusion in the paired analysis cohort. Of 56 patients initially screened between August 2024 and February 2025, one patient receiving FLU/CFA non-myeloablative conditioning was excluded due to a distinct toxicity profile. Of the 55 remaining patients, 47 had paired stool samples at both T0 (admission, pre-conditioning) and T1 (day of graft infusion) and constituted the primary analysis cohort. The cohort was then stratified into two analytic groups: chemotherapy-only regimens (TT/FLU/MEL and FLU/MEL; n = 34) and TBI-containing regimens (FLU/TBI and FLU/MEL/TBI; n = 13).

We conducted a prospective, single-center, observational longitudinal study at the Bone Marrow Transplant Department of Fundeni Clinical Institute (Bucharest, Romania). Adult patients (≥18 years) consecutively admitted for allo-HSCT between August 2024 and February 2025 were eligible for enrollment, regardless of primary diagnosis, donor type, or conditioning intensity. Patients were excluded if they were unable or unwilling to provide written informed consent, had a pre-existing diagnosis of inflammatory bowel disease, or were unable to provide a fecal sample at both relevant time points. A total of 56 patients were initially enrolled. One patient receiving a fludarabine/cyclophosphamide non-myeloablative (FLU/CFA) regimen was excluded because of its distinct toxicity profile and because it represents a single case, leaving 55 patients available for inclusion. Of these, 47 had paired stool samples available at both pre-conditioning admission (T0) and on the day of graft infusion (T1), forming the primary analysis cohort. The CONSORT-style patient flow diagram is presented in Figure 1.

Donor type was classified according to high-resolution HLA matching at HLA-A, -B, -C, -DRB1, and -DQB1 loci: matched unrelated donors (MUD) were defined as 10/10 matched; mismatched unrelated donors (MMUD) had at least one allele mismatch (9/10 or less); matched related donors (MRD) were 10/10 HLA-identical siblings; haploidentical donors (HAPLO) were related donors sharing one HLA haplotype.

Per institutional protocol, all patients received antifungal prophylaxis with a second-generation azole, antiviral prophylaxis with acyclovir, and gut decontamination with rifaximin, all of which were commenced at the start of conditioning. Systemic broad-spectrum antibacterial prophylaxis was initiated at the start of conditioning. Systemic broad-spectrum antibacterial prophylaxis was initiated only at the onset of neutropenia, after T1.

### Fecal sample collection

Fecal samples were collected longitudinally at five pre-specified time points within the institutional research protocol: at hospital admission, before starting conditioning therapy (T0); on the day of stem cell infusion (T1, day 0); 30 days after transplantation (T2); 100 days after transplantation (T3); and at the onset of biopsy-proven gastrointestinal acute graft-versus-host disease (Tx), when applicable. The present analysis focuses exclusively on the paired T0 and T1 samples, which capture the immediate effect of the conditioning regimen on the gut microbial community before the onset of aplasia, systemic broad-spectrum antibacterial prophylaxis, and mucositis. Samples were collected by patients into sterile containers (approximately 1–2 g of stool per sample), immediately frozen at −20 °C, transferred to the laboratory within 48 hours, and stored at −80 °C until DNA extraction. All samples included in this analysis successfully passed DNA extraction and 16S rRNA sequencing quality control.

### DNA extraction and 16S rRNA gene sequencing

Total genomic DNA was extracted from fecal samples using the QIAamp PowerFecal Pro DNA Kit (Qiagen, Hilden, Germany) according to the manufacturer’s protocol. DNA concentration and purity were assessed by NanoDrop spectrophotometry and Qubit fluorometry. The V4–V5 hypervariable region of the bacterial 16S rRNA gene was amplified using the 515F and 944R primers [14]. Library preparation, indexing PCR with the Nextera XT Index Kit v2 (Illumina), and sequencing on an Illumina MiSeqDx platform with v3 chemistry (2 × 300 bp paired-end reads) were performed as previously described [14]. Negative extraction controls were included throughout the workflow to monitor for contamination. Raw sequencing reads were demultiplexed using the Casava software package (Illumina).

### Bioinformatic analysis

Raw sequencing reads were processed using QIIME 2 [15]. Quality filtering, denoising, and chimera removal were performed using DADA2 [16], generating amplicon sequence variants (ASVs) at single-nucleotide resolution. Taxonomic classification was assigned using a Naïve Bayes classifier trained on the V4–V5 region of the Greengenes 13_8 reference database (released August 2013) [17], with a confidence threshold of 0.70. All diversity metrics were computed using the QIIME diversity core-metrics-phylogenetic function on the rarefied feature table at a sequencing depth of 1,334 reads per sample, selected to retain all evaluable samples while ensuring that rarefaction curves had reached a plateau. Alpha diversity was quantified primarily using the Shannon diversity index, reflecting both species richness and evenness; secondary alpha-diversity metrics (Simpson index, observed ASVs, Faith’s phylogenetic diversity) were obtained from the bioinformatic pipeline and are reported descriptively in the supplementary technical report. Beta diversity was assessed using the Bray-Curtis dissimilarity index and visualized via principal coordinates analysis (PCoA). Differences in community composition between T0 and T1 were tested using permutational multivariate analysis of variance (PERMANOVA) with 9,999 permutations, and homogeneity of multivariate dispersion was assessed using the betadisper function.

### Statistical analysis

The primary endpoint was the change in Shannon diversity between T0 and T1. Secondary endpoints included the magnitude of decline stratified by conditioning regimen type, conditioning intensity (RIC vs. MAC), and number of cytotoxic agents (2 vs. 3 agents). Continuous variables were reported as medians and interquartile ranges (Q1–Q3); categorical variables were expressed as frequencies and percentages. The Wilcoxon signed-rank test was used to compare Shannon diversity between T0 and T1, both across the overall cohort and within prespecified subgroups. The Mann−Whitney U test was used to compare the change in Shannon diversity (Δ Shannon = T1 − T0) between independent groups defined by regimen type, intensity, or number of agents. The Kruskal−Wallis H test was used to compare Δ Shannon across the four conditioning regimens simultaneously. Effect sizes for paired analyses were calculated as r = |Z| / √N. All *P* values are two-sided; statistical significance was set at *P* < 0.05. Statistical analyses were performed using IBM SPSS Statistics version 29; bioinformatic processing and beta-diversity analyses were performed using QIIME 2 [15] and R version 4.5.2 (vegan package for PERMANOVA).

### Clinical data collection

Clinical and demographic data were prospectively collected from the institutional electronic health records, including age, sex, primary diagnosis, time from diagnosis to transplantation, donor type, conditioning regimen and components (with specific doses), conditioning intensity classification (RIC vs. MAC according to EBMT criteria), and GVHD prophylaxis regimen.

## Results

### Study population

Demographic and clinical characteristics of the paired analysis cohort are summarized in Table 1. The median age at transplantation was 45.0 years (Q1–Q3: 36.0–54.0; range 19.8–64.5), and 27 patients (57.4%) were male. The majority of patients had a diagnosis of acute leukemia (36 patients, 76.6%), including acute myeloid leukemia (AML), acute lymphoblastic leukemia (ALL), and mixed-lineage acute leukemia, with the remaining 11 patients (23.4%) presenting with other hematologic malignancies (chronic myeloid leukemia, non-Hodgkin lymphoma, myelodysplastic syndrome, or myeloproliferative neoplasm). Median time from diagnosis to transplantation was 11.0 months (Q1–Q3: 8.4–16.5). Donor types were distributed as follows: matched unrelated donor (MUD, *n* = 16; 34.0%), mismatched unrelated donor (MMUD, *n* = 14; 29.8%), matched related donor (MRD, *n* = 9; 19.1%), and haploidentical donor (HAPLO, *n* = 8; 17.0%). All patients received peripheral blood stem cells as the graft source (*n* = 47; 100%). GVHD prophylaxis consisted predominantly of post-transplant cyclophosphamide combined with mycophenolate mofetil and a calcineurin inhibitor (PTCy + MMF + CNIs) in 45 patients (95.7%); the remaining two patients (4.3%) received methotrexate plus a calcineurin inhibitor (MTX + CNIs).

**Table 1:** Demographic and clinical characteristics of the paired analysis cohort (*n* = 47)

Variable	Patient cohort (*n* = 47)
**DEMOGRAPHICS**	
Age at transplant (years), median (Q1–Q3)	45.0 (36.0–54.0)
Age range (min–max), years	19.8–64.5
Sex, *n* (%)	
Male	27 (57.4%)
Female	20 (42.6%)
**PRIMARY DIAGNOSIS**	
Acute leukemia (AML, ALL, mixed-lineage), *n* (%)	36 (76.6%)
Other hematologic disease, *n* (%)	11 (23.4%)
Time from diagnosis to transplant (months), median (Q1–Q3)	11.0 (8.4–16.5)
**DONOR AND GRAFT**	
Donor type, *n* (%)	
Matched unrelated donor (MUD)	16 (34.0%)
Mismatched unrelated donor (MMUD)	14 (29.8%)
Matched related donor (MRD)	9 (19.1%)
Haploidentical donor (HAPLO)	8 (17.0%)
Graft source: peripheral blood stem cells (PBSC), *n* (%)	47 (100%)
**GvHD PROPHYLAXIS**	
PTCy + MMF + CNIs, *n* (%)	45 (95.7%)
MTX + CNIs, *n* (%)	2 (4.3%)

Abbreviations: PTCy, post-transplant cyclophosphamide; MMF, mycophenolate mofetil; CNIs, calcineurin inhibitors; MTX, methotrexate; Q1–Q3, first to third quartile.

### Conditioning regimens

The 47 patients in the paired analysis cohort received four distinct conditioning regimens, all fludarabine-based, with or without melphalan, thiotepa, and total body irradiation (Table 2). The most frequent regimen was thiotepa/fludarabine/melphalan (TT/FLU/MEL), administered to 20 patients (42.6%), followed by fludarabine/melphalan (FLU/MEL) in 14 patients (29.8%), fludarabine/TBI (FLU/TBI) in eight patients (17.0%), and fludarabine/melphalan/low-dose TBI (FLU/MEL/TBI) in five patients (10.6%). For analytic purposes, regimens were grouped into two categories: chemotherapy-only regimens (TT/FLU/MEL and FLU/MEL; *n* = 34; 72.3%) and TBI-containing regimens (FLU/TBI and FLU/MEL/TBI; *n* = 13; 27.7%).

**Table 2:** Conditioning regimens used in the paired analysis cohort (*n* = 47), with breakdown by intensity category (RIC vs. MAC) and analysis group (chemotherapy-only vs. TBI-containing)

Conditioning regimen	*n* (%)	Components	Analysis group	RIC	MAC
**TT/FLU/MEL**	20 (42.6%)	Thiotepa + Fludarabine + Melphalan	Chemo-only	18	2
**FLU/MEL**	14 (29.8%)	Fludarabine + Melphalan	Chemo-only	14	0
**FLU/TBI**	8 (17.0%)	Fludarabine + TBI	TBI-containing	5	3
**FLU/MEL/TBI**	5 (10.6%)	Fludarabine + Melphalan + low-dose TBI	TBI-containing	5	0
**TOTAL**	**47 (100%)**	**—**	**—**	**42 (89.4%)**	**5 (10.6%)**

Abbreviations: TT, Thiotepa; FLU, Fludarabine; MEL, Melphalan; TBI, Total Body Irradiation; RIC, Reduced-Intensity Conditioning; MAC, Myeloablative Conditioning.

According to EBMT intensity criteria, 42 patients (89.4%) received reduced-intensity conditioning (RIC), and five patients (10.6%) received myeloablative conditioning (MAC). The five MAC patients comprised two recipients of TT/FLU/MEL (one of whom received thiotepa at a double standard dose of 10 mg/kg) and three recipients of FLU/TBI with a TBI dose of 12 Gy. Notably, the TBI-containing group had a higher proportion of MAC patients than the chemotherapy-only group (23.1% vs. 5.9%), consistent with the standard classification of high-dose TBI regimens as myeloablative.

### Overall Change in Alpha Diversity Between T0 and T1

**Figure 2:**
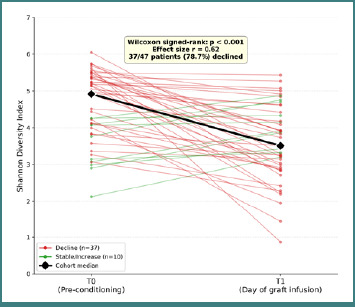
Paired Shannon diversity index at T0 (pre-conditioning) and T1 (day of graft infusion) in the analysis cohort (*n* = 47). Individual patient trajectories are shown as connecting lines; red lines indicate patients with decreased diversity at T1 (*n* = 37; 78.7%), and green lines indicate patients with stable or increased diversity (*n* = 10; 21.3%). The bold black line represents the cohort median trajectory. Wilcoxon signed-rank test: *P* < 0.001; effect size r = 0.62.

In the paired-analysis cohort (*n* = 47), Shannon diversity decreased substantially from admission (T0) to the day of graft infusion (T1). Median Shannon diversity was 4.91 (Q1–Q3: 3.83–5.37) at T0 and 3.51 (Q1–Q3: 2.92–4.42) at T1. The Wilcoxon signed-rank test confirmed a highly significant within-patient decline (Z = −4.275, *P* < 0.001), with a large effect size (r = 0.62). A decrease in Shannon diversity was observed in 37 of 47 patients (78.7%), while 10 patients (21.3%) showed stable or increased diversity at T1 (Table 3, Figure 2).

**Table 3:** Summary of statistical analyses of Shannon diversity changes from pre-conditioning (T0) to graft infusion (T1) in the paired analysis cohort (*n* = 47)

Analysis	Test	Comparison	*n*	Shannon T0 Median (Q1–Q3)	Shannon T1 / Δ Median (Q1–Q3)	Test statistic	*P* value	Effect size r
**OVERALL COHORT**								
**Primary**	Wilcoxon paired	T0 vs. T1	47	4.91 (3.83–5.37)	3.51 (2.92–4.42)	Z = −4.275	< 0.001	0.62
**STRATIFIED COMPARISONS — between-group differences in** Δ **Shannon (T1 − T0)**								
**By regimen type**	Mann–Whitney U	Chemo-only vs. TBI-containing	34 vs. 13	—	−0.86 vs. −0.43 (Δ)	U = 203	0.67	—
**By intensity**	Mann–Whitney U	RIC vs. MAC	42 vs. 5	—	−0.81 vs. −0.64 (Δ)	U = 102	0.92	—
**By number of agents**	Mann–Whitney U	2 agents vs. 3 agents	22 vs. 25	—	−0.48 vs. −0.92 (Δ)	U = 246	0.54	—
**By specific regimen**	Kruskal–Wallis H	4 regimens	47	—	— (Δ)	H = 1.345 (df = 3)	0.72	—
**WITHIN-SUBGROUP ANALYSES — Wilcoxon paired T0 vs. T1**								
**By regimen**	Wilcoxon paired	TT/FLU/MEL	20	4.48 (3.97–5.30)	3.33 (2.91–4.25)	Z = −2.651	0.008	0.61
	Wilcoxon paired	FLU/MEL	14	4.91 (3.85–5.49)	3.62 (2.46–4.62)	Z = −2.480	0.013	0.66
	Wilcoxon paired	FLU/TBI	8	5.21 (3.36–5.43)	3.69 (3.28–4.95)	Z = −1.400	0.161	0.50
	Wilcoxon paired	FLU/MEL/TBI	5	4.91 (4.22–5.04)	4.09 (2.99–4.15)	Z = −1.753	0.080	0.78
**By intensity**	Wilcoxon paired	RIC	42	4.90 (3.95–5.36)	3.62 (2.87–4.47)	Z = −4.057	< 0.001	0.63
	Wilcoxon paired	MAC	5	5.21 (3.22–5.47)	3.48 (3.09–4.41)	Z = −1.214	0.225	0.54

Abbreviations: TT, Thiotepa; FLU, Fludarabine; MEL, Melphalan; TBI, Total Body Irradiation; RIC, Reduced-Intensity Conditioning; MAC, Myeloablative Conditioning; Q1–Q3, first to third quartile. Statistical methods: Wilcoxon signed-rank test for paired comparisons (T0 vs. T1 within-subgroup); Mann–Whitney U test for between-group comparisons on Δ Shannon (T1 − T0); Kruskal–Wallis H test for comparisons across more than two groups. Effect size r calculated as |Z| / √N.

### Stratified between-group comparisons (Δ Shannon)

To evaluate whether the magnitude of conditioning-induced injury differed by regimen characteristics, the change in Shannon diversity (Δ Shannon = T1 − T0) was compared between subgroups using the Mann−Whitney U test (Table 3).

**Figure 3:**
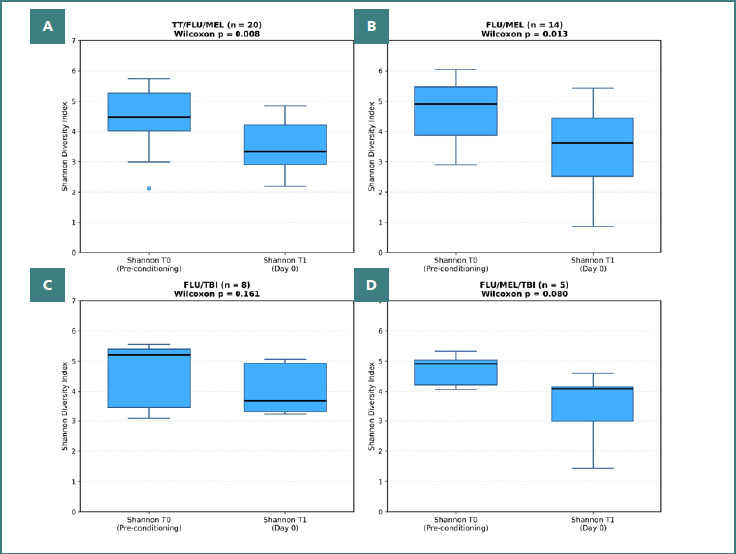
Paired Shannon diversity at T0 (pre-conditioning) and T1 (day of graft infusion) stratified by the four conditioning regimens. (A) TT/FLU/MEL (*n* = 20; Wilcoxon *P* = 0.008). (B) FLU/MEL (*n* = 14; Wilcoxon *P* = 0.013). (C) FLU/TBI (*n* = 8; Wilcoxon *P* = 0.161). (D) FLU/MEL/TBI (*n* = 5; Wilcoxon *P* = 0.080). Boxplots display medians and interquartile ranges. The within-subgroup decline is directionally consistent across all four regimens; statistical significance is reached in the two largest subgroups (panels A and B). The Y-axis is unified across panels (0–7) to facilitate direct visual comparison.

No significant difference in Δ Shannon was observed between the chemotherapy-only and TBI-containing analysis groups (median −0.86 vs. −0.43; U = 203, *P* = 0.67). Similarly, no significant difference was observed between the RIC and MAC subgroups (median −0.81 vs. −0.64; U = 102, *P* = 0.92) or between regimens using two versus three cytotoxic agents (median −0.48 vs. −0.92; U = 246, *P* = 0.54). The Kruskal−Wallis H test, comparing Δ Shannon across all four conditioning regimens simultaneously, also yielded a non-significant result (H = 1.345, df = 3, *P* = 0.72) (Figure 3).

### Within-subgroup paired analyses (Wilcoxon T0 vs. T1)

Within-subgroup Wilcoxon signed-rank tests confirmed a directionally consistent decline in Shannon diversity across all subgroups, with statistical significance reached in the four largest subgroups (Table 3).

**Figure 4:**
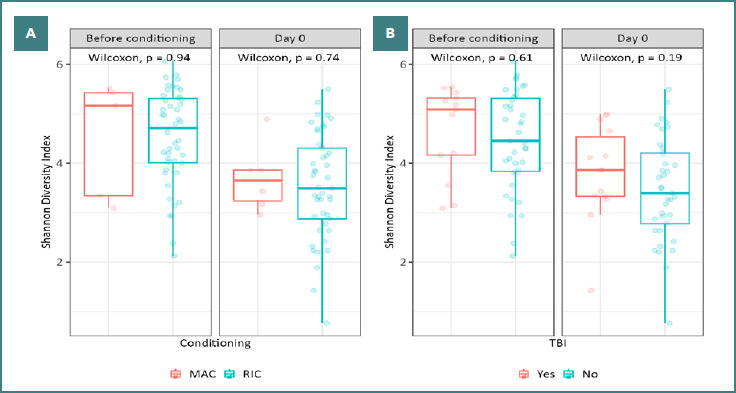
Shannon diversity index at T0 (before conditioning) and T1 (day 0) stratified by (A) conditioning intensity (MAC vs. RIC; Wilcoxon *P* = 0.94 at T0; *P* = 0.74 at T1) and (B) TBI exposure (yes vs. no; Wilcoxon *P* = 0.61 at T0; *P* = 0.19 at T1). Boxplots display medians and interquartile ranges; jittered points represent individual patients. Between-group comparisons did not reach statistical significance at either timepoint, supporting the reproducibility of the conditioning-induced injury across stratification dimensions.

Stratification by specific regimen demonstrated significant decline in TT/FLU/MEL (*n* = 20; Z = −2.651, *P* = 0.008; r = 0.61) and FLU/MEL (*n* = 14; Z = −2.480, *P* = 0.013; r = 0.66), and non-significant trends in FLU/TBI (*n* = 8; Z = −1.400, *P* = 0.161; r = 0.50) and FLU/MEL/TBI (*n* = 5; Z = −1.753, *P* = 0.080; r = 0.78) (Figure 3). Stratification by conditioning intensity demonstrated a highly significant decline in RIC patients (*n* = 42; Z = −4.057, *P* < 0.001; r = 0.63), while the MAC subgroup did not reach statistical significance (*n* = 5; Z = −1.214, *P* = 0.225; r = 0.54), reflecting the small sample size (Figure 4A).

### Beta diversity

**Figure 5:**
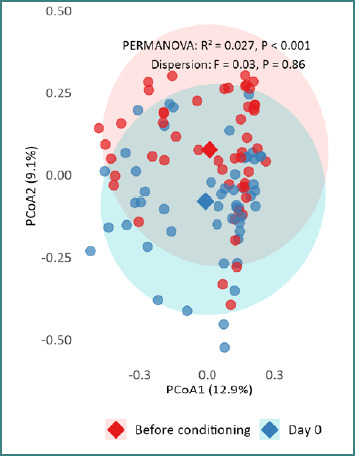
Principal coordinates analysis (PCoA) of Bray-Curtis dissimilarity between T0 (red) and T1 (blue) samples. PERMANOVA test for differences in community composition: R^2^ = 0.027, *P* < 0.001. Test for homogeneity of multivariate dispersion (betadisper): F = 0.03, *P* = 0.86. The significant compositional shift, in the absence of differential dispersion, indicates that conditioning produces a coherent change in microbial community structure rather than a simple increase in between-patient variability.

Beta diversity, assessed using Bray-Curtis dissimilarities and visualized with principal coordinates analysis (PCoA), showed a significant shift in microbial community composition between T0 and T1 (PERMANOVA *P* < 0.001), with the first two principal coordinates capturing the dominant axes of variation. Importantly, a test for homogeneity of multivariate dispersion (betadisper) yielded a non-significant result (*P* = 0.86), indicating that the observed compositional differences reflect a true shift in community structure rather than a change in within-group variability (Figure 5).

## Discussion

In this prospective single-center cohort of 47 adult allo-HSCT recipients, we demonstrate that conditioning produces a substantial, statistically robust decline in gut microbial Shannon diversity between admission (T0) and the day of graft infusion (T1). The magnitude of the effect — a median decrease of approximately 1.4 log-equivalent units, with a large standardized effect size (r = 0.62) and decline observed in nearly four-fifths of patients — establishes conditioning as a discrete and quantifiable insult on the gut microbial community, occurring before the contributions of post-conditioning aplasia, broad-spectrum antibiotic prophylaxis, and mucositis can confound the interpretation.

The most important methodological contribution of this study is the demonstration that the conditioning-induced microbiota injury is reproducible across three independent stratification dimensions: agent type (chemotherapy vs. TBI-containing), conditioning intensity (RIC vs. MAC), and number of cytotoxic agents (2 vs. 3). None of these between-group comparisons yielded statistical significance, with *P* values consistently above 0.5, and the four within-regimen Wilcoxon tests showed directionally consistent decline regardless of subgroup composition. The convergence of these analyses supports the interpretation that conditioning, considered as a category of intervention, constitutes a coherent biological injury to the gut microbial community, with the specific drug or radiation modality playing a secondary role relative to the overall intensity of cytotoxic stress.

Our findings are consistent with — and extend — the conditioning-associated microbiota injury (CMBI) framework developed by Shouval and colleagues, which stratified more than 1,000 patients into three injury tiers based on alpha-diversity loss [11]. While that retrospective multicenter analysis included a broader range of regimens, including high-intensity busulfan/cyclophosphamide and TBI/cyclophosphamide combinations not represented in our cohort, our results demonstrate that even within a cohort dominated by reduced-intensity regimens (89.4% RIC), the same coherent pattern of diversity loss is recoverable. Notably, busulfan was absent from our cohort, reflecting institutional practice favoring melphalan- and TBI-based platforms. The absence of significant differences between our chemotherapy-only and TBI-containing groups (Mann–Whitney *P* = 0.67) contrasts with the larger-scale CMBI gradient reported by Shouval [11], but is compatible with the recent observation by Rashidi *et al*. that TBI- and chemotherapy-based conditioning produce regimen-specific microbial signatures rather than purely quantitative differences in injury magnitude [13].

The absence of a statistically significant difference between RIC and MAC subgroups (*P* = 0.92) is biologically informative, although it must be interpreted with caution given the small MAC subgroup (*n* = 5). While clinical trials comparing RIC and MAC have established intensity as a determinant of transplant-related mortality, relapse, and overall survival in patients with AML, MDS, and other hematological malignancies [18,19], our data suggest that intensity may not be the principal driver of the immediate (T0 → T1) microbial response. One mechanistic interpretation is that the rate-limiting injury at this early timepoint is the cumulative cytotoxic burden delivered over the conditioning window, which is comparable across modern regimens regardless of whether intensity is augmented by radiation or by additional alkylating agents. The TBI-containing group in our cohort also contained a higher proportion of MAC patients (23.1% vs. 5.9% in the chemotherapy-only group), reflecting institutional practice in which high-dose TBI (≥12 Gy) defines myeloablative intensity; this partial confounding between intensity and TBI exposure is a recognized limitation of single-center analyses.

A central conceptual contribution of this work is the framing of T1 — the day of stem cell infusion, after completion of conditioning but before the onset of aplasia-associated factors — as a discrete biological window for assessing microbiota status. At T0, the gut microbial community reflects the cumulative effects of prior antineoplastic therapy, antibiotic exposure, hospitalization, and pre-existing comorbidity [4,8]. At T1, this baseline is overlaid by the acute effect of conditioning alone: melphalan and thiotepa are administered early in the conditioning window, while TBI is typically delivered in the final days, with all components fully captured by the time of graft infusion. After T1, the trajectory of the microbiota becomes dominated by secondary insults — neutropenia, fluoroquinolone prophylaxis, broad-spectrum antibiotics for infections, parenteral nutrition, and mucositis — which cumulatively reshape the community in ways that are difficult to disentangle. T1 therefore represents a methodologically clean reference point for quantifying the direct contribution of conditioning and a potentially actionable target for protective interventions, including prebiotic supplementation, fecal microbiota transplantation, or engineered consortia [6,7,20-22].

The beta diversity analysis confirmed that the conditioning-associated decline in alpha diversity is accompanied by a meaningful shift in community composition, with a statistically significant PERMANOVA result (*P* < 0.001) and no evidence of differential dispersion (betadisper *P* = 0.86). This indicates that conditioning does not merely homogenize or destabilize the microbial community but produces a coherent compositional change, consistent with the taxonomic shifts toward Enterococcus dominance and loss of commensal anaerobes described in larger cohorts [11,23].

### Strengths and limitations

The principal strengths of this study include its prospective design with protocolized sampling, the use of paired pre- and post-conditioning samples within each patient (which eliminates between-patient confounding), uniform sample processing and bioinformatic analysis, and statistical validation in SPSS with parallel methods to ensure reproducibility. The single-institution setting ensures consistent clinical practice across the cohort, particularly regarding GVHD prophylaxis (PTCy-based in 95.7% of patients) and supportive care.

Several limitations should be acknowledged. First, the sample size of 47 paired patients, while adequate for the primary endpoint, is limited for detecting subtle between-group differences in stratified analyses. The MAC subgroup (*n* = 5) and the FLU/MEL/TBI subgroup (*n* = 5) are particularly small, and within-subgroup Wilcoxon tests for these groups, while directionally concordant, did not reach statistical significance. The medium-to-large effect sizes observed in these small subgroups (r = 0.50–0.78) suggest that the lack of statistical significance reflects limited power rather than absence of effect. Second, our cohort is dominated by RIC regimens (89.4%), reflecting contemporary institutional practice but limiting generalizability to centers with a higher proportion of myeloablative or busulfan-based regimens. Third, busulfan-containing regimens are not represented in our cohort, which precludes direct comparison with the broader CMBI literature [11,12]. Fourth, the rarefaction depth used for diversity computation (1,334 reads per sample) was selected to retain all evaluable samples and to provide robust estimates of the Shannon index, but it may underestimate rare-taxon contributions; secondary alpha-diversity metrics (Simpson, observed ASVs, Faith’s PD) are reported descriptively rather than as primary outcomes. Fifth, taxonomic classification relied on the Greengenes 13_8 reference database, which has not been actively maintained since 2013. More recently curated references, including SILVA (release 138 or later) and Greengenes2, offer improved taxonomic resolution and phylogenetic accuracy; however, because our primary and secondary end-point were alpha- and beta-diversity metrics derived from ASV-level data rather than fine-grained taxonomic assignments, the choice of reference database is unlikely to have materially affected the central findings, and future taxonomic analysis from this cohort will employ an updated reference. Finally, this analysis focuses exclusively on the T0 → T1 transition and does not address downstream clinical outcomes such as engraftment kinetics, GVHD, infection, or transplant-related mortality, which will be the subject of future analyses leveraging the full longitudinal sampling protocol.

## Conclusion

In this prospective cohort of 47 adult allo-HSCT recipients, conditioning produces a substantial, statistically robust decline in gut microbial Shannon diversity between admission and the day of graft infusion, observed in nearly four-fifths of patients and reproducible across three independent stratification dimensions (regimen type, intensity, and number of agents). The convergence of these analyses supports the interpretation of conditioning as a coherent biological insult on the gut microbiota, independent of the specific cytotoxic modality. The day of stem cell infusion (T1) represents a biologically meaningful and methodologically clean reference point for quantifying conditioning-induced microbiota injury and a potentially actionable window for microbiota-protective interventions in allo-HSCT recipients. Future research should focus on linking T1 microbiota status to downstream clinical outcomes and evaluating the efficacy of pre- and peri-conditioning interventions designed to preserve microbial diversity at this critical time point.

## Data Availability

The 16S rRNA gene sequencing data and associated metadata generated and analyzed during this study are not publicly deposited at the time of submission due to ongoing patient privacy and institutional data-sharing review. The data are available from the corresponding author upon reasonable request, subject to approval by the Ethics Committee of Fundeni Clinical Institute.

## Personal thanks

The authors gratefully acknowledge the patients who participated in this study, as well as the medical and nursing staff of the Bone Marrow Transplant Department of Fundeni Clinical Institute for their dedicated care of the patients and their support throughout the study.
